# Supplementary material for: Prevalence, Microbiological Profile, and Risk Factors of Healthcare-Associated Infections in Intensive Care Units: A Retrospective Study in Aljouf, Saudi Arabia
Source: Microorganisms. 2025 Aug 17;13(8):1916. doi: 10.3390/microorganisms13081916 (PMC12388808; doi:10.3390/microorganisms13081916)
Supplement: Supplementary file 1 [file microorganisms-13-01916-s001.zip › microorganisms-3800857-supplementary.pdf]

Table S1: Microbiological profile of patients with healthcare-associated infections (HAIs), including causative agents, associated device, specimen type, and polymicrobial status (N = 40)

HAI Patient #	Organism (s) Detected per Patient	Polymicrobial	Associated Device	Specimen Type for HAI	Causative Agent	HAI Type
**1**	*Candida albicans*	No	Urinary Catheter	Urine	*Candida albicans*	CAUTI
**2**	*Klebsiella pneumoniae*	No	Urinary Catheter	Urine	*Klebsiella pneumoniae*	CAUTI
**3**	*Staphylococcus aureus* + *Klebsiella pneumoniae*	Yes	Urinary Catheter	Urine	*Staphylococcus aureus*	CAUTI
**4**	*Providencia stuartii*	No	Urinary Catheter	Urine	*Providencia stuartii*	CAUTI
**5**	*Acinetobacter baumannii*	No	Urinary Catheter	Urine	*Acinetobacter baumannii*	CAUTI
**6**	*Candida auris*	No	Urinary Catheter	Urine	*Candida auris*	CAUTI
**7**	*Klebsiella pneumoniae* + *Pseudomonas spp*	Yes	Urinary Catheter	Urine	*Klebsiella pneumoniae*	CAUTI
**8**	*Klebsiella pneumoniae*	No	Urinary Catheter	Urine	*Klebsiella pneumoniae*	CAUTI
**9**	*Klebsiella pneumoniae* + *Proteus mirabilis*	Yes	Urinary Catheter	Urine	*Klebsiella pneumoniae*	CAUTI
**10**	*Pseudomonas spp*	No	Urinary Catheter	Urine	*Pseudomonas spp*	CAUTI
**11**	*Escherichia coli*	No	Urinary Catheter	Urine	*Escherichia coli*	CAUTI
**12**	*Candida albicans*	No	Urinary Catheter	Urine	*Candida albicans*	CAUTI
**13**	*Candida tropicalis*	No	Urinary Catheter	Urine	*Candida tropicalis*	CAUTI

**14**	*Klebsiella pneumoniae*	No	Urinary Catheter	Urine	*Klebsiella pneumoniae*	CAUTI
**15**	*Pseudomonas spp + Proteus mirabilis*	Yes	Urinary Catheter	Urine	*Pseudomonas spp*	CAUTI
**16**	*Klebsiella pneumoniae + Enterococcus spp*	Yes	Urinary Catheter	Urine	*Klebsiella pneumoniae*	CAUTI
**17**	*Acinetobacter baumannii*	No	Urinary Catheter	Urine	*Acinetobacter baumannii*	CAUTI
**18**	*Serratia marcences*	No	Urinary Catheter	Urine	*Serratia marcences*	CAUTI
**19**	*Klebsiella pneumoniae*	No	Urinary Catheter	Urine	*Klebsiella pneumoniae*	CAUTI
**20**	*Klebsiella pneumoniae*	No	Central Line	Blood	*Klebsiella pneumoniae*	CLABSI
**21**	*Acinetobacter baumannii*	No	Central Line	Blood	*Acinetobacter baumannii*	CLABSI
**22**	*Klebsiella pneumoniae*	No	Central Line	Blood	*Klebsiella pneumoniae*	CLABSI
**23**	*Acinetobacter baumannii + Klebsiella pneumoniae*	Yes	Central Line	Blood	*Acinetobacter baumannii*	CLABSI
**24**	*Klebsiella pneumoniae*	No	Central Line	Blood	*Klebsiella pneumoniae*	CLABSI
**25**	*Acinetobacter baumannii + Klebsiella pneumoniae*	Yes	Central Line	Blood	*Acinetobacter baumannii*	CLABSI
**26**	*Klebsiella pneumoniae*	No	Central Line	Blood	*Klebsiella pneumoniae*	CLABSI
**27**	*Acinetobacter baumannii*	No	Mechanical Ventilator	Sputum	*Acinetobacter baumannii*	VAP
**28**	*Nil growth (IVAC)*	No	Mechanical Ventilator	Sputum	*Nil growth (IVAC)*	VAP
**29**	*Acinetobacter baumannii*	No	Mechanical Ventilator	Sputum	*Acinetobacter baumannii*	VAP
**30**	*Acinetobacter baumannii*	No	Mechanical Ventilator	Sputum	*Acinetobacter baumannii*	VAP

**31**	*Providencia stuartii*	No	Mechanical Ventilator	Sputum	*Providencia stuartii*	VAP
**32**	*Acinetobacter baumannii* + *Klebsiella pneumoniae*	Yes	Mechanical Ventilator	Sputum	*Acinetobacter baumannii*	VAP
**33**	*Pseudomonas spp*	No	Mechanical Ventilator	Tracheal Wash	*Pseudomonas spp*	VAP
**34**	*Klebsiella pneumoniae* + *Acinetobacter baumannii*	Yes	Mechanical Ventilator	Sputum	*Klebsiella pneumoniae*	VAP
**35**	*Klebsiella pneumoniae*	No	Mechanical Ventilator	Tracheal Wash	*Klebsiella pneumoniae*	VAP
**36**	*Acinetobacter baumannii*	No	Mechanical Ventilator	Sputum	*Acinetobacter baumannii*	VAP
**37**	*Pseudomonas spp*	No	Mechanical Ventilator	Sputum	*Pseudomonas spp*	VAP
**38**	*Acinetobacter baumannii*	No	Mechanical Ventilator	Sputum	*Acinetobacter baumannii*	VAP
**39**	*Klebsiella pneumoniae*	No	Mechanical Ventilator	Sputum	*Klebsiella pneumoniae*	VAP
**40**	*Proteus mirabilis*	No	Mechanical Ventilator	Sputum	*Proteus mirabilis*	VAP
